# The health consequences of politically-induced trauma

**DOI:** 10.1371/journal.pmen.0000579

**Published:** 2026-03-20

**Authors:** Ralph I. Horwitz

**Affiliations:** Aging +Cardiovascular Discovery Center, Temple University, Philadelphia, Pennsylvania, United States of America; PLOS: Public Library of Science, UNITED KINGDOM OF GREAT BRITAIN AND NORTHERN IRELAND

The relationship between lived political experience and human biology is well established in the academic literature, yet the same harmful patterns recur across time and regions, suggesting that this knowledge, however well documented, has yet to be adequately translated into policy or practice. Current events in American cities offer a timely context in which to shine a spotlight on this known relationship. Large-scale immigration enforcement operations involving the detention of adults and children, family separation, and the visible presence of armed federal agents in residential neighborhoods have created conditions of sustained civic unrest whose physiological consequences extend far beyond their immediate experiences. This Opinion argues that such circumstances constitute a substantial public health concern, one whose burden will manifest across years and decades, while also noting that communities organizing peaceful collective responses to adversity may simultaneously generate biological as well as social forms of resilience.

## The biological architecture of trauma

The pathway from adverse political experience to physiological pathology is well-mapped in the scientific literature. When children witness the detention of family members, when parents observe their households disrupted by enforcement action, when communities see neighbors removed into federal custody, the body responds with a cascade of biological changes that begin immediately and may persist for a lifetime [1].

We have known for decades that upon experiencing trauma, the hypothalamic-pituitary-adrenal (HPA) axis, the system regulating the body’s stress responses, becomes dysregulated, flooding the system with cortisol in patterns that exceed adaptive capacity. This stress hormone, adaptive in acute doses, becomes physiologically toxic when sustained. Neural circuits reorganize accordingly: the amygdala becomes hyperresponsive while the prefrontal cortex, responsible for executive function and emotional regulation, shows decreased activity and, in chronic cases, measurable structural atrophy. These are not metaphorical changes. They are visible on neuroimaging and quantifiable through hormonal assays [2]. The immune system becomes exhausted under conditions of chronic stress, with pro-inflammatory cytokines circulating at elevated levels and accelerating virtually every chronic disease process [3]. Telomeres show accelerated shortening under sustained stress [4]. Epigenetic modifications, particularly genes governing stress-response pathways, may be transmitted to subsequent generations, meaning the biological consequences of today’s trauma may be expressed in the children and grandchildren of those affected [5,6].

The Adverse Childhood Experiences (ACE) literature has documented a dose-response relationship between childhood trauma and adult morbidity and mortality across nearly every organ system [7]. A child detained, separated from caregivers, or held in unfamiliar environments by armed agents accumulates ACE scores that predict cardiovascular risk decades later, increased likelihood of autoimmune disease, and vulnerability to mental health conditions. These are not speculative harms. The creation of future disease burden is observable in real time.

## A population-level health event

What distinguishes politically-induced trauma from other forms of acute stress is its collective nature and its capacity to radiate outward through communities well beyond direct participants. Enforcement-related unrest is not contained: its traumatic impact extends to witnesses, bystanders, and to those not physically proximate but who closely identify with those affected. Direct participants suffer most severely, but witnessing itself is traumatic. Neighbors observe enforcement convoys. Children see other children removed. Families understand viscerally that their own circumstances are not categorically different. A climate of diffuse apprehension develops in which hypervigilance becomes the baseline state and the capacity for physiological recovery is compromised.

The cumulative effect is population-level trauma whose public health implications are comparable in scale to those of natural disasters or acute mass trauma, but with the critical distinction that it is sustained over months or years. Chronic stress loads are consistently more harmful to biological systems than equivalent acute loads. This pattern is not unique to the United States. Comparable dynamics have been documented across diverse global contexts from conflict-displaced populations in the Middle East and sub-Saharan Africa to European refugee communities where exposure to state violence and forced migration generates ACE burdens whose downstream health consequences are equivalent [8,9]. The biology of childhood adversity does not respect state borders.

## Disease pathways

The biological disruptions represent causal mechanisms with well-characterized downstream effects. Post-traumatic stress disorder (PTSD), which emerges at elevated rates in communities subject to sustained civic unrest, is not simply a psychiatric diagnosis: it generates a pro-inflammatory biological state that substantially increases risk for autoimmune diseases including rheumatoid arthritis, inflammatory bowel disease, and multiple sclerosis [10]. The hypervigilance, intrusive recall, and sleep disruption that characterize PTSD have physiological manifestations that actively accelerate disease pathogenesis. A Swedish cohort study of over 100,000 individuals found that those with stress-related disorders were approximately 30–40% more likely to be subsequently diagnosed with autoimmune diseases, compared with matched unexposed individuals and unaffected siblings, a finding that persisted after adjustment for genetic confounding [10].

Cardiovascular disease risk rises sharply with chronic stress and trauma exposure. Sustained elevation of cortisol and catecholamines, the primary stress hormones released by the adrenal glands, combined with chronic inflammation and endothelial dysfunction, contributes to accelerated atherosclerosis, hypertension, and increased incidence of myocardial infarction and stroke [11]. Metabolic disruption follows similar patterns, with trauma exposure predicting elevated rates of obesity, type 2 diabetes, and metabolic syndrome.

Particularly relevant for clinicians is the finding that trauma affects not only disease development but treatment response. PTSD-related immune dysregulation with elevated pro-inflammatory cytokines, altered glucocorticoid sensitivity, and accelerated immune senescence creates treatment-resistant phenotypes that can mitigate conventional medical interventions. For instance, a patient whose rheumatoid arthritis develops in the context of sustained enforcement-related fear may respond less well to immune-mediated therapy than one whose disease arose from genetic predisposition alone [12]. Children now accumulating ACE scores through enforcement-related disruption and family separation will carry these biological imprints into adulthood, shaping their health trajectories for decades.

## The biology of collective response

Biology is not destiny, and human beings are not passive recipients of harm. Alongside the substantial evidence for the pathological consequences of politically-induced trauma, a growing body of research documents that purposeful, collective action may confer measurable biological as well as psychological benefits.

The distinction between hedonic and eudaimonic wellbeing is central here. Hedonic wellbeing experienced as pleasure, comfort, and the absence of distress is one form of positive health-relevant experience. Eudaimonic wellbeing, by contrast, is derived from living in accordance with one’s values, engaging in purposeful activity, and contributing to something larger than oneself. Research on the conserved transcriptional response to adversity (CTRA), a pattern of gene expression characterized by increased inflammatory activity and decreased antiviral responses, has demonstrated that eudaimonic wellbeing produces powerful biological signatures, modifying gene expression in ways that reduce inflammatory activity and enhance antiviral immune function even under conditions of ongoing stress [13]. Those who engage in organized, non-violent responses such as legal advocacy, community protection networks, and mutual aid structures are not merely participating in political activity. They are engaging in forms of meaning-making that may confer biological protection against some of the harms they face. Social connection and perceived social support reduce cortisol reactivity and pro-inflammatory cytokine production. Collective purpose activates biological pathways that are more robustly protective under adversarial conditions than those engaged by hedonic pleasure alone [14].

This is emphatically not an argument that resistance is required for health, or that those who do not organize are in some sense biologically unprotected through any failure of their own. Such logic would perversely place the burden of biological protection on those already carrying the greatest burden of exposure. Nor does collective action erase or fully mitigate the harms of sustained trauma. But clinicians and public health practitioners should recognize that communities under sustained unrest are not only accumulating damage. Both truths must coexist in our understanding: politically-induced trauma causes biological harm, and purposeful collective response may generate biological resilience.

## Bearing witness

The fundamental principle at stake has deep roots in the social determinants of health literature: biography becomes biology. The conditions to which populations are exposed inscribe themselves into bodies and are expressed as disease. Government policies that generate sustained conditions of fear and civic unrest in civilian populations are pathogenic. The ACE literature, the neuroendocrinology of chronic stress, the CTRA framework, and the epidemiology of trauma-associated disease converge on this conclusion. Documenting that relationship is a legitimate and necessary professional responsibility.

At the same time, we must bear witness to the resilience that communities generate while navigating these conditions and recognize the collective action that may provide biological protection even as it pursues moral and political objectives. The full picture requires both. What is happening on the streets of American cities will be written into the biology of those exposed, and physicians will be treating the consequences for generations. That is not a political claim. It is a medical one [15].
